# Mild Hypothermia Therapy for Moderate or Severe Hypoxicischemic Encephalopathy in Neonates

**Published:** 2018-01

**Authors:** Weihua LIAO, Huiying XU, Jing DING, Hong HUANG

**Affiliations:** Dept. of Neonatology, Nanfang Hospital, Southern Medical University, Guangzhou, China

**Keywords:** Hypoxic-ischemic encephalopathy, Neonates, Mild hypothermia, Nursing

## Abstract

**Background:**

To study the nursing method of mild hypothermia therapy for moderate or severe hypoxic-ischemic encephalopathy (HIE) in neonates.

**Methods:**

According to the inclusion and exclusion criteria, 48 patients were selected from Nanfang Hospital from December 2015 to October 2016 and randomly divided into the control group (n=24) and observation group (n=24). The control group received routine treatment and nursing, while the observation group received the same treatment as the control group combined with mild hypothermia treatment and nursing. The clinical effects were compared.

**Results:**

The total effective rate in the observation group was increased and mortality was decreased, and the differences were statistically significant (*P*=0.029 and 0.033, respectively). The 28 d neonatal behavioral neurological assessment and nursing satisfaction scores in the observation group were higher than those in the control group, and the differences were statistically significant (*P*=0.017 and 0.008, respectively).

**Conclusion:**

Mild hypothermia therapy for moderate or severe HIE in neonates is safe and effective, and the correct nursing method is important for guaranteeing proper clinical treatment.

## Introduction

Hypoxic-ischemic encephalopathy (HIE) in neonates is primarily caused by perinatal asphyxia, and has an incidence of about 0.5–3.0% (1). It is related to abnormal cerebral blood flow, disorder of energy and substance metabolism, inflammation, oxidative stress, apoptosis, and necrosis of brain tissue (2). It often occurs 6–12 h after childbirth, and symptomatic treatment is commonly used clinically (3).

Basic research has confirmed that (4) every 1°C drop in body temperature equates to a decrease of oxygen consumption by 5%. Mild hypothermia therapy is more effective for protecting nerves and promoting neural rehabilitation (5). It is safe and effective (6).

In this study, mild hypothermia therapy was conducted in neonates with moderate or severe HIE, and improved nursing management was applied, thereby achieving good clinical effects.

## Methods

### Patients

A total of 48 neonates diagnosed with neonatal asphyxia in Nanfang Hospital, Guangzhou, Guangdong, China from December 2015 to October 2016 were consecutively selected. Newborns were considered for active whole-body therapeutic hypothermia (TH) if they met a combination of diagnostic criteria including clinical, laboratory and amplitude-integrated electroencephalogram (aEEG) findings, as established by the first two large published trials of therapeutic hypothermia for neonatal HIE.

The criteria for treatment within 6 hours after birth included 1) gestational age ≥36 weeks and birth weight ≥ 2000g; 2) evidence of fetal distress including history of acute perinatal event; 3) evidence of neonatal distress as shown by at least one of the following: Apgar score ≦ 5 at 10 minutes, pH ≦ 7.0 within 1 hour of birth or base deficit < 16 mEq/L, or need for ventilation for at least 10 min after birth; and eligible infants were then assessed for evidence of moderate or severe encephalopathy by a certified examiner including lethargy, stupor, or coma, with one or more of hypotonia, abnormal reflexes (Oculomotor or pupillary abnormalities), an absent or weak suck, or clinical evidence of seizures.

The exclusion criteria included evidence of metabolic disease, congenital infection, major malformations, alcohol or drug embryopathies, hydrops, chromosome abnormalities, and MRI/CT evidence of long-standing brain damage or developmental abnormality. Patients were randomly divided into the control group (n=24) and treatment group (n=24). In the control group, there were 15 males and nine females, with gestational age of 36–39 weeks (average: 37.8 ± 2.2 weeks) and weight of 2.5–3.3 kg (average: 2.8 ± 0.5 kg). There were 17 cases of moderate HIE and seven cases of severe HIE. In the treatment group, there were 14 males and 10 females, with gestational age of 36–39.5 weeks (average: 38.2 ± 2.6 weeks) and weight of 2.5–3.2 kg (average 2.6 ± 0.7 kg). There were 16 cases of moderate HIE and eight cases of severe HIE. The baseline parameters of the two groups were comparable. This study was approved by the Ethics Committee of Nanfang Hospital. Signed written informed consents were obtained from the patients’ guardians.

### Research methods

The control group received routine treatment and nursing, mainly including oxygen uptake, sedation, neural nutrition, neural rehabilitation, normal blood perfusion, and management of blood pressure, blood glucose and electrolytes. The nursing procedures included monitoring of vital signs, checking for nervous system reactions, understanding changes of disease in a timely manner; paying attention to maintaining warm temperature, milk intake, urine and excrement, and jaundice; keeping the respiratory tract unobstructed, and improving rehabilitation training, such as for auditory, tactual, and visual stimulation.

The treatment group received the same treatment as the control group, combined with mild hypothermia treatment and nursing. The main treatment and nursing procedures were as follows: the instrument for treatment was a BLANKETROL III water-blanket medical temperature controller (CSZ, USA). For treatment, a far infrared rays radial type rescue table was used; the temperature switch for radiation stage or the incubator was turned off, and the patient was placed under normal temperature. The room temperature was controlled at about 23 °C. Before treatment, at least two venous channels were established in patients if there was need for rescue when the state of disease changed. If the body temperature of neonates reached the acceptable range for mild hypothermia therapy, maintenance treatment was conducted directly. If not, maintenance treatment was induced, ensuring that the target temperature (33.5–34°C) for mild hypothermia therapy was reached within 1–2 h. Next, maintenance treatment was performed for neonates who reached the target temperature for mild hypothermia therapy. First, the temperature of the skin, nasopharynx, and esophagus was continuously monitored and recorded every 15 min until 1 h after reaching the target temperature; the temperature was then recorded every 2 h, and recorded every 1 h during the temperature recovery period. If the body temperature of neonates was 1 °C below or above the target temperature, and the neonates were agitated and trembling, the physician was notified immediately. The skin of neonates was checked once every 4 h, and their position was changed once every 2 h.

For blood gas analysis during treatment, the temperature at the time was noted, and corresponding symptomatic and supportive treatment were provided according to the actual clinical needs of the neonates. The conventional treatment time was 72 h. During treatment, physicians should try to avoid unnecessary stimulation to neonates, cleaned the respiratory secretions as required, and turned neonates over on time. Ideally, the respiratory rate of patients was slower and with smooth rhythms, but with different frequency. Nodding-like breathing and decreased breathing extent suggested over-inhibition of the respiratory center, and the temperature was timely adjusted. When the disease was stable, patients had a red complexion with warm limbs and steady, strong pulse. With the drop in blood pressure, pale face, and acromegaly, the temperature was raised to correct the electrolyte and acid-base balance. Micropump 24 h nutritional supplementation could be combined to prevent necrotizing enterocolitis in neonates. It was also needed to prevent cold injury syndrome and edema. The skin was massaged regularly. The temperature recovery process was slow using the natural or artificial temperature recovery method. The temperature was raised by 0.5 °C every 2 h, and the rectal temperature was recorded every 1 h until the temperature rose to 36.5 °C.

### Observation indexes

The total effective rate, mortality, and incidence of complications between the two groups were compared. In addition, the 28 d neonatal behavioral neurological assessment (NBNA) and nursing satisfaction scores were compared. If the laboratory examination results returned to normal reference ranges, the disease was cured; if the clinical symptoms were improved significantly and were stable, and the laboratory examination results were improved by ≥50%, the curative effect was significant; if the clinical symptoms were improved, and the laboratory examination results were improved by 30%–50%, the treatment was effective; otherwise, the treatment was ineffective. In terms of NBNA score, normal was 37–40 points, suspicious was 35–36 points, and abnormal was below 35 points. Nursing satisfaction was evaluated using a reliability and validity questionnaire scale designed by our department, including four aspects: nursing skills, work attitude, problem solving condition, and treatment effect, with 25 points each. Higher score indicated higher satisfaction.

### Statistical analysis

SPSS20.0 software (Chicago, IL, USA) was used for data analysis. Numerical data are presented as mean ± standard deviation, and the independent sample *t*-test was used for intergroup comparisons; categorical data are presented as case or percentage (%), and a *χ*^2^-test was used for intergroup comparisons; *P*<0.05 was taken as statistically significant.

## Results

In the treatment group, the total effective rate was increased and mortality was decreased, and the differences were statistically significant (*P*=0.029 and 0.033, respectively) (Table 1).

**Table 1:** Comparisons of total effective rate and mortality (cases (%))

***Group***	***Case***	***Cured***	***Significant***	***Effective***	***Ineffective***	***Total effective rate***	***Mortality***
Control group	24	2	6	5	11	13 (54.2)	8 (33.3)
Treatment group	24	7	8	5	4	20 (83.3)	2 (8.3)
*χ*^2^						4.752	4.547
*P*						0.029	0.033

In the control group, there was one case of severe cerebral injury, two cases of infection, and one case of multiple organ dysfunction. The total incidence of complications was 16.7%. In the treatment group, there was one case of cold injury syndrome, one case of respiratory depression, and one case of infection. The total incidence of complications was 12.5%. There was no difference in the incidence of complications between the two groups (*χ*^2^=0.000, *P*=1.000).

The 28 d NBNA and nursing satisfaction scores of the treatment group were higher than those of the control group, and the differences were statistically significant (*P*=0.017 and 0.008, respectively) (Table 2).

**Table 2:** Comparisons of NBNA and nursing satisfaction scores

***Group***	***Case***	***NBNA***	***Abnormal nerve function (cases (%))***	***Nursing satisfaction score***
Control group	24	35.8±2.5	9 (37.5)	68.9±14.5
Treatment group	24	37.2±2.3	3 (12.5)	85.6±12.3
*t*/*χ*^2^		5.214	4.000	5.629
*P*		0.017	0.046	0.008

## Discussion

At present, the operational experience of mild hypothermia therapy for moderate or severe HIE is different in each center. The nursing management of temperature reduction and recovery is not unified, and treatment effects differ. However, appropriate mild hypothermia therapy is effective for reducing brain damage and promoting the recovery of neurological function (7). Mild hypothermia therapy is characterized by simple operation and safe application for term infants, although it is dangerous for infants aged less than 36 weeks (8). The rectal temperature (33.5–34 °C) is often taken as the temperature control standard; the breathing, heart rate, blood pressure, and blood gas indexes of pediatric patients are closely monitored to prevent hypothermia, scleredema, infection, and other complications (9). When the rectal temperature drops to the minimum acceptable value (33°C), the switch for the incubator or FIR radiating type bed is turned on to maintain the temperature and avoid excessive vascular contraction (10). The temperature recovery process is also important, and should not be too fast, so as to avoid coagulation disorders, rebound hyperkalemia, hypovolemic shock, and other complications (11).

Detailed and scientific mild hypothermia nursing is an important factor to guarantee the treatment effect and improve the prognosis. Nursing procedures are applied throughout the course of mild hypothermia therapy, from the instructions given before the operation until the full preparation, temperature regulation, and monitoring of vital signs. Symptomatic treatment is provided for breathing, digestion, and for the neural, urinary, and circulatory systems (12). Nursing personnel must understand the principles and matters requiring attention during mild hypothermia therapy. They must be familiar with the structure and method of application of the temperature control therapeutic apparatus, master various first aid methods, have an increased sense of responsibility in their work, and identify changes in disease in time, and report them to the physician, so that symptomatic treatment can be provided (13). Proper care is important for improving the rehabilitation of neurological function, promoting healthy development, and increasing immunity (14).

The present study showed that in the treatment group, the total effective rate was increased, mortality was decreased, and there was no difference in the comparison of the incidence of complications. The 28 d NBNA and nursing satisfaction scores in the treatment group were higher than those in the control group. If moderate or severe HIE in neonates is not treated correctly and in a timely manner, it will often cause permanent brain damage, hypophrenia, cerebral palsy, epilepsy and other sequelae. Furthermore, it can increase the incidence of infection and multiple organ dysfunction, and seriously reduce the patient’s quality of life (15). Mild hypothermia reduces oxidative stress, inflammation, apoptosis, and necrosis. In addition, it promotes recovery of neural function, inhibits the accumulation of lactic acid and various toxins, and reduces brain injury, primarily by decreasing the demand of brain cells for oxygen (16).

This study had a small sample-size. The total effective rate of treatment was 83.3%, and the mortality rate was 8.3%. The symptoms mainly included cold injury syndrome, respiratory depression, and infection. Therefore, the reasonable control of temperature was key for clinical treatment (17). Under the premise of standards for unified temperature control, the severity of the patient’s illness, laboratory indexes, and individual differences, it is necessary to reasonably adjust the range of temperature, and closely monitor the change in each system to maximize the success rate of treatment (18). The short-term and long-term effects of mild hypothermia treatment also require further validation. The 28 d NBNA is a sensitive index reflecting the short-term treatment effect, and the score reflects the patient’s long-term development of intelligence (19). Nursing satisfaction is important for the evaluation of nursing work. Patients with moderate or severe HIE are often treated in the ICU, and the visiting hours of their parents are limited. Therefore the worry, anxiety, and other negative emotions of parents are also important factors influencing the treatment effect. Creating a favorable environment in the ward, guaranteeing the emergency handling capacity, and maintaining good communication with parents, are important parameters in the assessment of nursing satisfaction (20).

## Conclusion

Mild hypothermia therapy for moderate or severe HIE in neonates is safe and effective, and correct nursing method helps guarantee effective clinical treatment.

## Ethical considerations

Ethical issues (Including plagiarism, informed consent, misconduct, data fabrication and/or falsification, double publication and/or submission, redundancy, etc.) have been completely observed by the authors.
